# Electrospun PCL/PGS Composite Fibers Incorporating Bioactive Glass Particles for Soft Tissue Engineering Applications

**DOI:** 10.3390/nano10050978

**Published:** 2020-05-19

**Authors:** Marina Luginina, Katharina Schuhladen, Roberto Orrú, Giacomo Cao, Aldo R. Boccaccini, Liliana Liverani

**Affiliations:** 1 Dipartimento di Ingegneria Meccanica, Chimica e dei Materiali, University of Cagliari, Via Marengo 2, 09123 Cagliari, Italy; m.luginina@gmail.com (M.L.); roberto.orru@dimcm.unica.it (R.O.); giacomo.cao@dimcm.unica.it (G.C.); 2 Institute of Biomaterials, Department of Materials Science and Engineering, University of Erlangen-Nuremberg, Cauerstr. 6, 91058 Erlangen, Germany; katharina.ks.schuhladen@fau.de (K.S.); aldo.boccaccini@fau.de (A.R.B.)

**Keywords:** poly(glycerol-sebacate), electrospinning, benign solvents, composite fibers, bioactive glass

## Abstract

Poly(glycerol-sebacate) (PGS) and poly(epsilon caprolactone) (PCL) have been widely investigated for biomedical applications in combination with the electrospinning process. Among others, one advantage of this blend is its suitability to be processed with benign solvents for electrospinning. In this work, the suitability of PGS/PCL polymers for the fabrication of composite fibers incorporating bioactive glass (BG) particles was investigated. Composite electrospun fibers containing silicate or borosilicate glass particles (13-93 and 13-93BS, respectively) were obtained and characterized. Neat PCL and PCL composite electrospun fibers were used as control to investigate the possible effect of the presence of PGS and the influence of the bioactive glass particles. In fact, with the addition of PGS an increase in the average fiber diameter was observed, while in all the composite fibers, the presence of BG particles induced an increase in the fiber diameter distribution, without changing significantly the average fiber diameter. Results confirmed that the blended fibers are hydrophilic, while the addition of BG particles does not affect fiber wettability. Degradation test and acellular bioactivity test highlight the release of the BG particles from all composite fibers, relevant for all applications related to therapeutic ion release, i.e., wound healing. Because of weak interface between the incorporated BG particles and the polymeric fibers, mechanical properties were not improved in the composite fibers. Promising results were obtained from preliminary biological tests for potential use of the developed mats for soft tissue engineering applications.

## 1. Introduction

The fabrication of synthetic, biodegradable scaffolds able to mimic native features of extracellular matrix (ECM) is a significant challenge for soft and hard tissue engineering (TE) [1]. Among the different scaffold fabrication techniques, electrospinning (ES) has emerged as a promising approach for different biomedical applications, since it provides a simple and versatile tool to produce interconnected porous nano- and microfibrous structures from various synthetic and natural polymers [2,3]. Briefly, the principle of ES is based on the application of high-intensity electrical potential between two electrodes of opposite polarity, a metallic needle containing the polymeric solution and a grounded target. ES is strongly affected by different parameters, which have been extensively investigated in the literature [2,4,5] and can be generally grouped in three main categories, namely solution features (e.g., concentration, viscosity, rheological properties, presence of particles in the suspension, etc.), process parameters (e.g., applied voltage, flow rate, needle diameter, distance between tip of the syringe and collector, type of collector and needle(s)), and external environmental conditions, such as relative humidity and temperature.

Poly(glycerol-sebacate) (PGS), a biocompatible and biodegradable soft elastomer first introduced by Wang and co-workers [6], has recently attracted much attention since its properties appeared to be particularly interesting for regenerative medicine. PGS has been studied for a broad range of applications including cardiovascular patches [7,8], heart valves [9], as well as for cartilage [10,11], bone [12], corneal [13] and nerve [14,15] tissue engineering, for the retina [16,17] and tympanic membrane healing [18,19]. Synthesis of PGS involves a two-step procedure: First, the PGS pre-polymer (PGS_p_) is synthesized from pre-polycondensation of glycerol and sebacic acid, then the cross-linked polymer can be obtained by additional vacuum heat treatment of the PGS_p_ precursor. The mechanical properties and degradation kinetics of PGS, which are relevant features for tissue engineering applications, can be tailored during the synthesis process by altering the molar ratio of glycerol and sebacic acid constituents, curing time and temperature. For example, Chen and co-workers studied the effect of curing temperature on the mechanical properties of PGS patches for the myocardial tissue healing [7]. It is also worth to mention that electrospinning of neat PGS represents a challenging task because of the low solution viscosity caused by the low molecular weight and chain entanglement of the polymer [6]. To increase its electrospinnability, PGS can be blended with other polymers, either synthetic or natural [20]. 

In this context, poly(ε-caprolactone) (PCL), a semi-crystalline biocompatible, non-toxic, and degradable polymer, which has been approved by the US Food and Drug Administration (FDA) for certain biomedical applications [21,22], is suitable to be processed by ES. Generally, PCL is used in the field of hard TE, where longer healing periods are required [23,24], because of its relative stability in vivo and lower degradation rate compared to PGS [25,26]. Blending PCL and PGS polymers allows the fabrication of electrospun fibrous scaffold with varying degradation behavior. Furthermore, the presence of the more stable PCL can ensure a better structural support for cells at the site of implantation, while faster degrading PGS provides the essential space for the new ECM deposition. Elastic PCL/PGS fiber mats with different constituent ratios have been already successfully tested as materials for heart valve regeneration [20,27,28]. Moreover, advanced electrospun PCL/PGS mats with patterned topographical features as well as PCL/PGS scaffolds functionalized with vascular endothelial growth factors have been developed for cardiac patch applications [29]. Furthermore, Kalakonda and co-workers studied antibacterial properties of PCL/PGS fibrous scaffold coated with silver for wound healing applications [30]. 

During the fabrication of PCL/PGS electrospun scaffolds, harsh solvents consisting of mixtures of anhydrous chloroform, dichloromethane, or dimethyl carbonate with ethanol or methanol are mainly used. It should be noted that an appropriate solvent selection plays a pivotal role for the obtainment of smooth, bead-free fibers by electrospinning. Recently, the substitution of the toxic solvents mentioned above with more environmental friendly benign solvents was attempted [31]. In this regard, acetic acid (AA), according to guidelines established by the International Conference on Harmonization of Technical Requirements for Registration of Pharmaceuticals for Human use (ICH), belongs to class 3 of solvents, which are considered less toxic and harmful for humans [32], and it could be considered as benign solvent for electrospinning, according to the definition given in a previous report [33]. Vogt et al. first reported on the possibility to obtain PCL/PGS (1:1) fibrous mats using AA for cardiac TE applications [8]. 

Recently, the scientific community has focused its attention on the use of bioactive glasses (BGs) also for soft tissue engineering applications [34,35]. Indeed, previous researches on BGs have been mainly oriented on the investigation of the osteogenic properties of these materials, important for the bone regeneration. After the discovery of the ability of BGs to promote angiogenesis [36], ascribed to the dissolution products arising from their contact with body fluids, more attention is being dedicated to applications related to wound healing [37,38,39]. Besides angiogenesis, the presence of specific ions in the BG, i.e., boron, has positive effects on the wound healing process [40]. In this context, the aim of the present research work was to fabricate electrospun PCL/PGS composite fibrous structures incorporating silicate (composition 13-93) and borosilicate (labelled as 13-93BS) bioactive glass particles as inorganic component. Acetic acid was utilized as benign solvent for electrospinning. Biocompatibility of electrospun mats with seven different compositions was examined as well as cell attachment and proliferation. The influence of fiber composition on the mechanical properties and degradation characteristics was also assessed.

## 2. Materials and Methods 

### 2.1. Solution Preparation 

Electrospun fiber mats with seven different compositions, as reported in Table 1, were fabricated starting from PCL (80 kDa, Sigma Aldrich, Munich, Germany) solutions. Glacial acetic acid (AA, VWR, Darmstadt, Germany) was utilized as a solvent. 

Synthesis of PGS polymer ((C_13_H_22_O_5_)_n_) was performed according to the protocol reported by Wang et al. [2]. Briefly, in order to obtain PGS prepolymer (PGS_p_), 0.1 M sebacic acid (99%, Sigma Aldrich, Munich, Germany) and 0.1 M polyol glycerol (BioXtra, 99%, Sigma Aldrich, Munich, Germany) were mixed and subsequently heated at 120 °C under inert nitrogen atmosphere for 24 h. Mildly cross-linked PGS polymer (PGS_mxl_) was further obtained from PGS_p_ precursor by additional treatment in an oven at 120 °C under vacuum (1.3–2.5 × 10^−2^ mTorr) for 24 h, as previously reported by Vogt et al. [8].

Silicate and borosilicate BG (13-93 and 13-93BS, respectively) utilized in the present study to fabricate composite electrospun mats were synthesized by the melt-quenching technique, according to the procedure described by Schuhladen et al. [41]. Briefly, starting reagents (all from Sigma-Aldrich^®^, Munich, Germany), were H_3_BO_3_, (CaHPO_4_)(2(H_2_O)), CaCO_3_, K_2_CO_3_, Na_2_CO_3_, MgO, and Belgian quartz sand. Melting was carried out in a platinum crucible under the following conditions: 1100 °C for 3 h (for BG 13-93BS) and 1360 °C for 3 h (for BG 13-93). Further, all compositions were cast and annealed at 520 °C. To ensure homogeneity, melting was performed twice. The BGs were then crushed using a Jaw Crusher (Retsch, Germany) and grounded to fine powder with average particle size of 5–20 μm, using a zirconia planetary ball mill (Retsch, Germany). The composition of both BGs is reported in Table 2.

After the optimization process, solution preparation was carried out according to the following procedure: a 20% *w/v* solution of PCL in acetic acid was prepared, stirred overnight at room temperature, and sonicated for 30 min prior to the addition of PGS. PGS_p_ or PGS_mxl_ (50 wt.% respect to PCL amount) were further added to the solution, stirred and sonicated for additional 30 min. BG powders, 13-93 or 13-93BS, were homogeneously dispersed (30 wt.% respect to PCL amount) in polymeric solution and stirred for 5 min. Electrospinning was performed immediately after solution preparation in order to avoid possible alterations of bioactive glass particles in contact with the solvent.

### 2.2. Electrospinning Process

Electrospun fiber mats were obtained using a commercially available setup (Starter Kit 40KV Web, Linari Engineering srl, Valpiana (GR), Italy). The utilized parameters of the electrospinning process as well as temperature and relative humidity are summarized in Table 1. 

### 2.3. Characterization

#### 2.3.1. Microstructure and Composition

Scanning electron microscopy (SEM) *(*FE*-*SEM-EDS, Auriga Base, Carl-Zeiss, Jena, Germany) was conducted for examining the microstructure and *morphology* of the series of electrospun mats. Prior to SEM analysis, the samples were sputtered with gold using a sputter coater (Q150T, Quorum Technologies Ltd., Darmstadt, Germany). Magnification was varied in the range from 1000 to 45,000×. The average fiber diameter was calculated using the Fiji 1.51 s analysis software (NIH, Bethesda, MD, USA) [42]. The measurement of the diameter of 30 randomly chosen fibers was performed for each sample.

FTIR spectroscopy analysis was carried out in attenuated total reflectance mode (ATR) (IRAffinity-1S, Shimadzu, Japan). For the analysis, 40 spectral scans in absorbance mode were averaged across the wavenumber range of 4000 to 400 cm^−1^ with a resolution of 4 cm^−1^.

#### 2.3.2. Mechanical Characterization

Mechanical properties of a set of fiber mats were evaluated by uniaxial tensile strength test (5960 Dual Column Tabletop Testing System, Instron^®^, Darmstadt, Germany). The measurements were performed at a crosshead speed of 10 mm/min using a 100 N load cell. In order to avoid any undesired stretching of fiber mats before the tensile test, specimens were cut into rectangular shape (3 mm × 20 mm) and arranged into paper frames (20 mm × 20 mm) [43]. Average values and standard deviations of Young’s modulus (E), ultimate tensile strength (UTS) and strain to failure (FS) were determined based on five measurements for each composition. 

#### 2.3.3. Wettability

Measurements of static water contact angle on fibrous specimens were conducted by the sessile drop method (DSA30, Krüss GmbH, Hamburg, Germany) in air. To this aim, drops of 3 μL deionized water were placed onto the fiber mats. At least five measurements were performed for each composition at room temperature. The data were collected and analyzed using the DSA software (DSA4 2.0, Krüss GmbH, Hamburg, Germany).

#### 2.3.4. In Vitro Acellular Bioactivity and Degradation Study

The acellular bioactivity of composite fiber mats was evaluated by immersion tests in simulated body fluid (SBF) solution, prepared according to Kokubo’s protocol [44]. In addition, the in vitro degradation study of electrospun fiber mats was carried out in phosphate buffered saline (PBS) (VWR International GmbH, Darmstadt, Germany). 

Sample triplicates were immersed in 16 mL of either SBF or PBS solution at 37 °C, respectively. Experiments were performed in a standard incubator (KS 4000 i Control, IKA^®^-Werke GmbH & Co. KG, Staufen, Germany) with mild shaking (82 rpm). During the incubation stage, pH was monitored and recorded at the following time points: 1 h, 2 h, 4 h, 1 day, 4, and 7 days. After 1 and 7 days, the samples were removed from the solution, rinsed with ultra-pure water, dried, and investigated by SEM and ATR-FTIR, to examine the changes in morphology and chemical composition. Falcon tubes containing solutions of SBF or PBS without any samples were incubated for the entire period of the experiment to ensure and control the solution stability overtime.

#### 2.3.5. Cell Culture

A biological assay was performed using bone marrow-derived stromal cells, ST-2, (Deutsche Sammlung von Mikroorganismen und Zellkulturen GmbH, Braunschweig, Germany). Before the seeding step, ST-2 cells were cultured in RPMI 1640 medium (Thermo Fisher Scientific), supplemented with 10% fetal bovine serum (Lonza) as well as 1% penicillin/streptomycin (Lonza), and incubated at 37 °C with 5% CO_2_. Briefly, prior to cell test, all samples were cut and fixed on a holder for 24-well plates (CellCrownTM, Scaffdex, Sigma). Treatment under UV light was carried out for 1 h to disinfect the specimens. ST-2 cells were drop seeded onto fixed fiber samples with a density of 2.5·10^5^ cells/mL in a droplet of 100 µL of RPMI media per sample and incubated for 15 min. Then, 1 mL of RPMI medium was added to each well. Cell viability, proliferation, and morphology after 1 and 7 days were evaluated according to the protocol reported by Liverani et al. [33].

After 1 and 7 days, cell proliferation and cytotoxicity were assessed using colorimetric Cell Counting Kit-8 (CCK-8, Sigma Aldrich) assay based on tetrazolium salt WST-8 (2-(2-methoxy-4-nitrophenyl)-3-(4-nitrophenyl)-5-(2,4-disulfophenyl)-2H-tetrazolium, monosodium salt). The reduction of WST-8 by cellular dehydrogenases to an orange formazan product was measured in absorbance mode at 450 nm by the plate reader (PHOmo microplate reader, Autobio Labtec Instruments Co. Ltd., Zhengzhou City, China).

Cell morphology and adhesion were investigated after 1 and 7 days after seeding. To this aim, samples were observed by fluorescence microscopy (Axio Scope A1, Zeiss). Seeded samples were stained with rhodamine phalloidin and DAPI (Thermo Fisher Scientific, Waltham, MA, USA), according to the protocol reported previously by Liverani et al. [33]. Briefly, sample’s fixation was carried out using a solution containing 1,4-piperazinediethanesulfonic acid buffer, ethylene glycol tetra-acetic acid, polyethylene glycol, paraformaldehyde, PBS, and sodium hydroxide (Sigma Aldrich, Munich, Germany). Then, the specimens were rinsed three times with PBS and immersed in a permeabilization buffer containing Triton X-100, sucrose, and PBS (Sigma Aldrich, Munich, Germany). Prior to characterization, fixed samples were stained using rhodamine phalloidin solution (8 µL/mL) and DAPI solution (1 µL/mL). 

#### 2.3.6. Statistics

All experimental data are presented as average values ± standard deviation. One-way analysis of variance (ANOVA, Origin) was used to determine the differences between groups with a probability defined as (* *p* < 0.05).

## 3. Results and Discussion

### 3.1. Fiber Morphology

**Figure 1:**
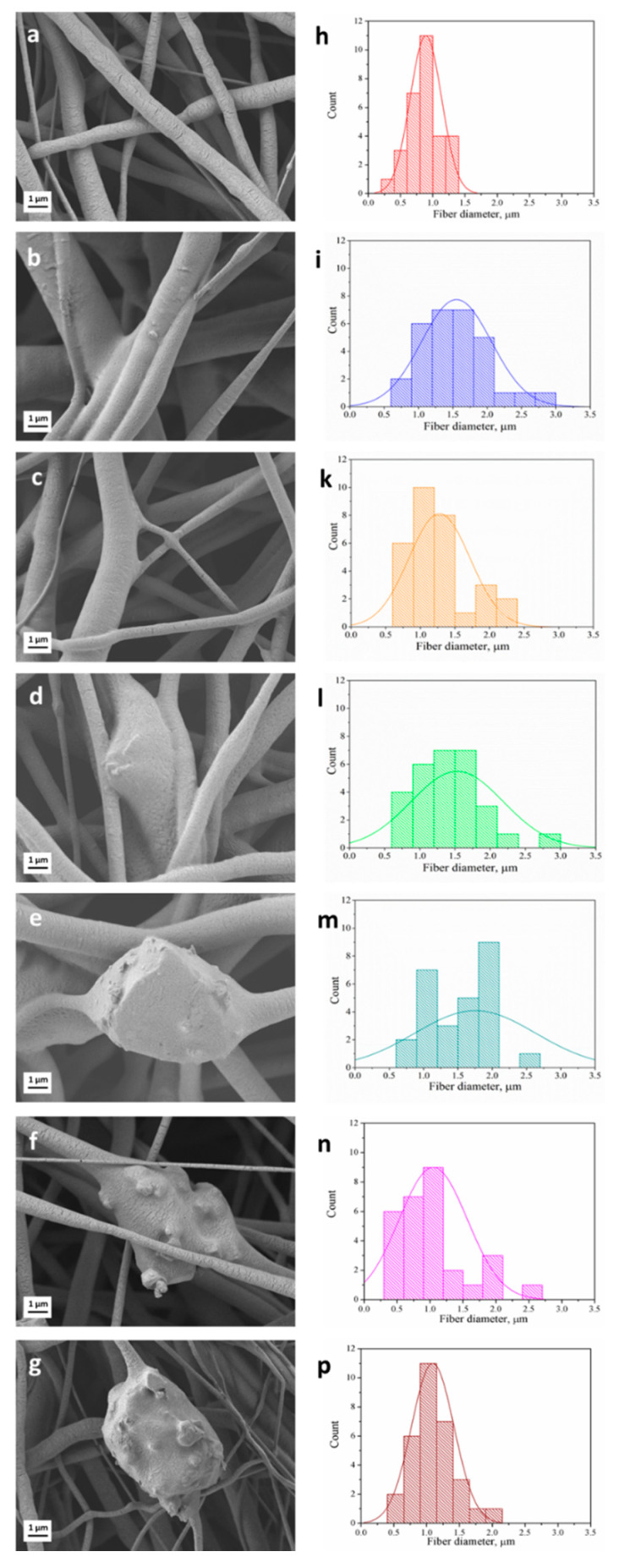
Scanning electron microscopy (SEM) micrographs of electrospun fiber mats and measured fiber diameter distribution: (**a**,**h**) Neat PCL (S5); (**b**,**i**) PCL/PGS_p_ (S1); (**c**,**k**) PCL/PGS_mxl_ (S2); (**d**,**l**) PCL/PGS_mxl_/13-93 (S3); (**e**,**m**) PCL/PGS_mxl_/13-93BS (S4); (**f**,**n**) PCL/13-93 (S6) and (**g**,**p**) PCL/13-93BS (S7), respectively.

SEM micrographs of the series of as-spun porous samples are shown in Figure 1a–g. In the present work, the selection of the key parameters for the electrospinning process, namely, the solution concentration and applied voltage, was adapted from a previous study [43]. It was shown that homogeneous, bead-free neat PCL fiber mats can be obtained using 20 *w/v*% solution of PCL in acetic acid and 15 kV applied voltage. The latter parameters as well as tip-target distance and flow rate (Table 1) were maintained constant in order to better clarify the influence of the composition, i.e., presence of PGS_p_ or PGS_mxl_ and BG particles on the mechanical and biological performances of fiber mats.

The average fiber diameter of electrospun mats of different compositions is reported in Table 3. It can be seen that the addition of PGS leads to an increase of the average fiber diameter. This phenomenon can be ascribed to the increasing total amount of polymer content in the solution. The value of the average fiber diameter for the neat PCL mats (0.9 ± 0.4 μm) falls within the range of 0.11–3.85 μm reported in the literature [33,43,45]. Moreover, the measured values of average fiber diameter for PCL/PGS_p_ (1.5 ± 0.5 μm) and PCL/PGS_mxl_ (1.5 ± 0.6 μm) are also in accordance with the literature, where the fiber diameter of PCL/PGS blends was reported to vary in the range between 0.55 and 4.7 μm [20,28,46,47]. Such feature demonstrates that the use of benign solvents did not affect the morphology of the electrospun composite fibers, whose average fiber diameter is comparable with the one obtained with standard (e.g., chloroform or dichloromethane) solvents for ES.

**Figure 2:**
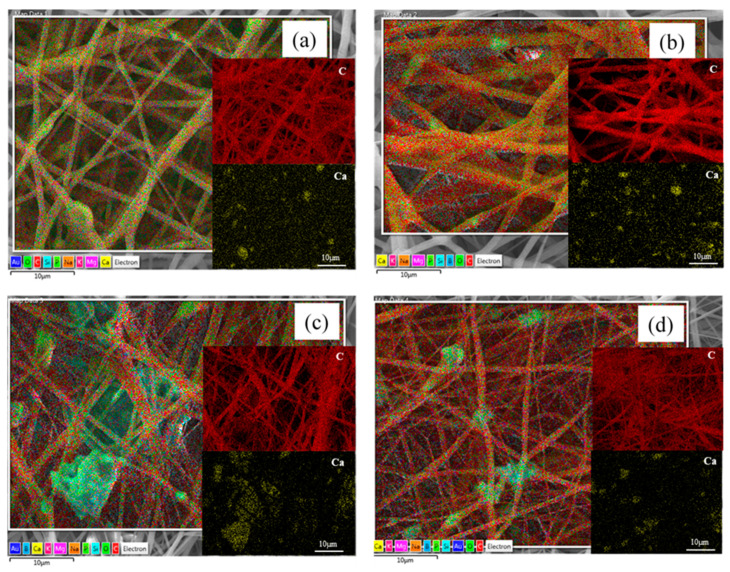
SEM/EDX micrographs of composite fibers, confirming the presence of BG particles in (**a**) PCL/PGSmxl/13-93 (S3), (**b**) PCL/PGSmxl/13-93BS (S4), (**c**) PCL/13-93 (S6), (**d**) PCL/13-93BS (S7) samples. In all micrographs two insets related to the mapping of carbon and calcium are reported.

The amount of BG added in the present work to fabricate composite fiber mats was fixed at 30 wt.% and maintained constant for all composite blends, in particular for PCL/13-93, PCL/13-93BS, PCL/PGS_mxl_/13-93, and PCL/PGS_mxl_/13-93BS. The incorporation of BG microparticles into the fibrous scaffolds was confirmed by the SEM/EDX analysis shown in Figure 1d–g and Figure 2. 

The addition of BG particles did not alter significantly the average fiber diameter (Table 3). However, the standard deviation in samples containing BG particles, i.e., PCL/13-93, PCL/13-93BS, PCL/PGS_mxl_/13-93, and PCL/PGS_mxl_/13-93BS, was found to increase in BG containing fibers. Moreover, the distribution of fiber diameter reported in Figure 1l–p shows that their maximum and minimum values vary more noticeably in samples containing BG particles. This effect can be likely caused by the increased suspension conductivity in presence of BG particles. 

### 3.2. Chemical Characterization 

**Figure 3:**
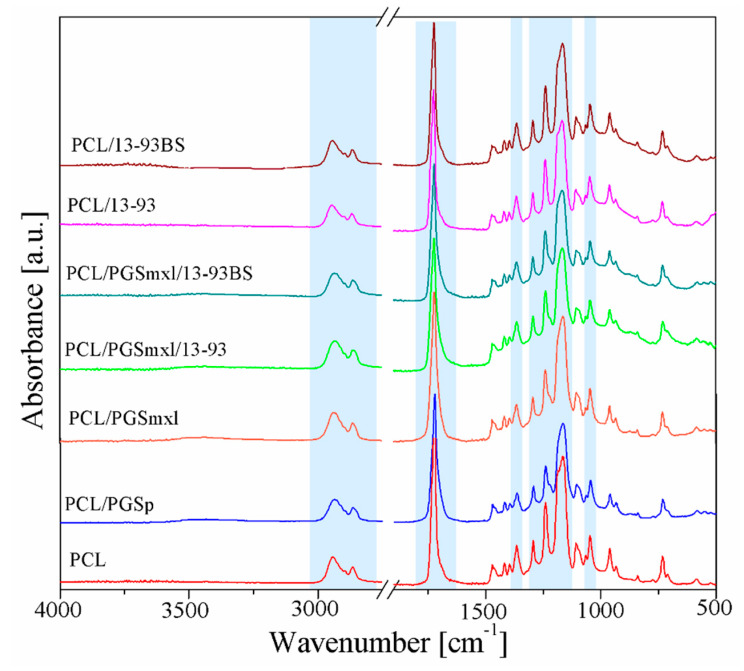
ATR-FTIR spectra of fiber mats in the wavenumber range 4000–500 cm^−1^. Main bands are indicated in light blue and discussed in detail in the text.

FTIR analysis was performed in order to examine the chemical composition of prepared fiber mats. Figure 3 illustrates the obtained spectra of neat PCL, PCL/PGSp, PCL/PGSmxl, and polymer/BG fiber composites. No typical absorption bands of acetic acid were detected, confirming that the solvent totally evaporated during the electrospinning process. 

All spectra exhibit the characteristic PCL bands already reported in the literature [8,46,47]: i.e., 2942 cm^−1^, 2865 cm^−1^, and 1366 cm^−1^, related to stretching of alkyl group (CH_2_); 1240 cm^−1^ and 1165 cm^−1^ peaks, attributed to symmetric and asymmetric C–O–C stretching, respectively; the peak centered around 1722 cm^−1^, associated to carbonyl stretching (C=O); finally, the peak around 1294 cm^−1^, due to the backbone C–O and C–C stretching. Several specific bands related to PGS and BG particles overlap the main PCL bands and therefore are not easily detectable in Figure 3.

PGS related vibration bands, namely at 2929 cm^−1^, 2851 cm^−1^, and 1384 cm^−1^, attributed to alkyl groups, the peak around 1734 cm^−1^, relative to carbonyl stretching (C=O), and C–O band stretching vibration at 1165 cm^−1^ [8], overlap with the PCL absorption bands. Moreover, the broad band between 3300 cm^−1^ and 2500 cm^−1^, corresponding to the stretch vibration of hydroxyl bond, cannot be distinguished since the amount of PCL is prevalent respect to PGS. Only a slight shift to lower the wavenumbers, typical for PGS, can be noticed when the spectra of PCL/PGS blends were analyzed. 

A similar situation can be observed when considering samples containing BG particles (PCL/13-93, PCL/13-93BS, PCL/PGS_mxl_/13-93, PCL/PGS_mxl_/13-93BS). Characteristic absorption bands of silicate 13-93 BG [41], namely Si–O–Si and Si–O stretching modes, located in the range 900–1100 cm^−1^, as well as those ones of Si–O–Si bending mode at 470 cm^−1^, are relatively weak and cannot be detected. Borosilicate 13-93BS BG exhibits the same vibrations displayed by silicate glass 13-93 along with additional peaks attributed to the B–O stretching mode of tetrahedral BO_4_ groups located around 700 cm^−1^ and in the range between 900–1100 cm^−1^; as well as B–O stretching bands of BO_3_ group situated in 1150–1300 cm^−1^ and 1200–1500 cm^−1^ ranges [41]. 

### 3.3. Wettability

Surface wettability or hydrophilicity of a biomaterial represents one of the key parameters which affect cell behavior via protein adsorption, platelet adhesion/activation, blood coagulation, and cell bacterial adhesion [48,49]. It has been reported that either strongly hydrophobic or hydrophilic surfaces are not favorable for supporting cell attachment. Indeed, the optimal water contact angle which allows effective cell adhesion is reported to be in the range between 40 and 70° [48,50].

In the present study, the wetting ability of as-spun fiber mats was evaluated by contact angle measurements. Fiber mats containing only PCL as polymer component, i.e., PCL, PCL/13-93, PCL/13-93BS, were found to exhibit hydrophobic behavior with contact angles of 100 ± 5°, 100 ± 4°, and 101 ± 4°, respectively. The estimated values lay in the range between 98 to 133° previously reported in the literature for PCL electrospun mats [8,20,46], confirming that the presence of BG particles and the related roughness of the composite mats do not affect their wettability.

On the contrary, blended fibers containing PGS, namely PCL/PGS_p_, PCL/PGS_mxl_, PCL/PGS_mxl_/13-93, and PCL/PGS_mxl_/13-93BS showed highly hydrophilic behavior. The contact angle was not detectable, since the water drop deposited on the surface of the samples rapidly penetrated and spread through all the tested compositions. Thus, being strongly hydrophilic because of the hydroxyl groups attached to its backbone, PGS strongly affects the wettability of PCL/PGS blend mats [20,46]. 

### 3.4. Degradation in PBS Solution 

**Figure 4:**
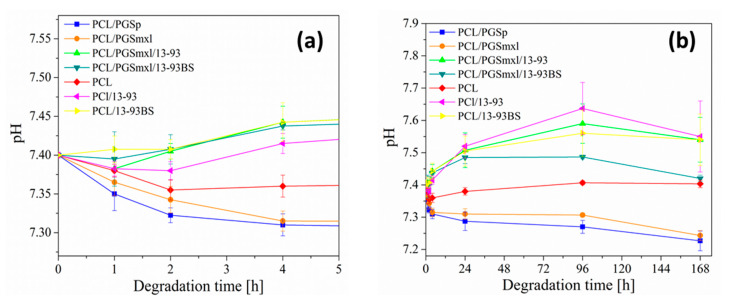
pH of medium during immersion test of samples up to (**a**) 5 h and (**b**) 168 h in phosphate buffered saline (PBS) at 37 °C.

It is known that, when exposed to biological environments, biomaterials undergo degradation, so that their initial physicochemical properties progressively change as function of time. In particular, in case of polymers, degradation leads to polymer chain scissions. For instance, PCL and PGS, which belong to aliphatic polyesters, are both subjected to hydrolytic degradation through the cleavage of ester linkages, but with different rates. Total resorption of PGS in vivo has been observed within few weeks after implantation [26,51]. On the other hand, PCL, being semi-crystalline at room temperature, is relatively stable in vivo and displays low resorption rate [25,52]. In addition, it has been found that both crystallinity and molecular weight of PCL strongly affect its degradation rate [25,53]. In this perspective, it is particularly interesting to study PCL/PGS blends, in order to obtain hybrid materials that exhibit modulated or tunable degradation. To investigate degradation features, neat PCL, PCL/PGS, and PCL/PGS/BG blends were subjected to in vitro immersion tests in PBS solution. Over the course of the test, the change of pH of the medium was monitored, in order to track possible modifications which could be related to BG ion release and PGS degradation. Measurements were performed after 1 h, 2 h, 4 h, 24 h, 96 h, and 168 h of incubation. The curves showing the corresponding pH changes are plotted in Figure 4a,b.

It can be observed that during the first 4 h, pH values of PBS solution in contact with PCL/PGS_p_ and PCL/PGS_mxl_ mats drop more significantly, indicating acidification of the medium; on the contrary, neat PCL samples led to only modest changes of pH. In addition, after 168 h of immersion, the pH of the solutions containing PCL/PGS_p_ and PCL/PGS_mxl_ was found to be 7.2 ± 0.3 and 7.2 ± 0.2, respectively. Acidification effect of PGS due to the leaching of non-reacted monomers has been reported in the literature [54,55]. In order to remove residual monomers and oligomers, which can be potentially harmful for cells, Jeffries et al. [54] washed the samples in a mixture of ethanol and water, while Chen et al. [55] conditioned them prior to cell seeding in a culture medium for 6 days.

**Figure 5:**
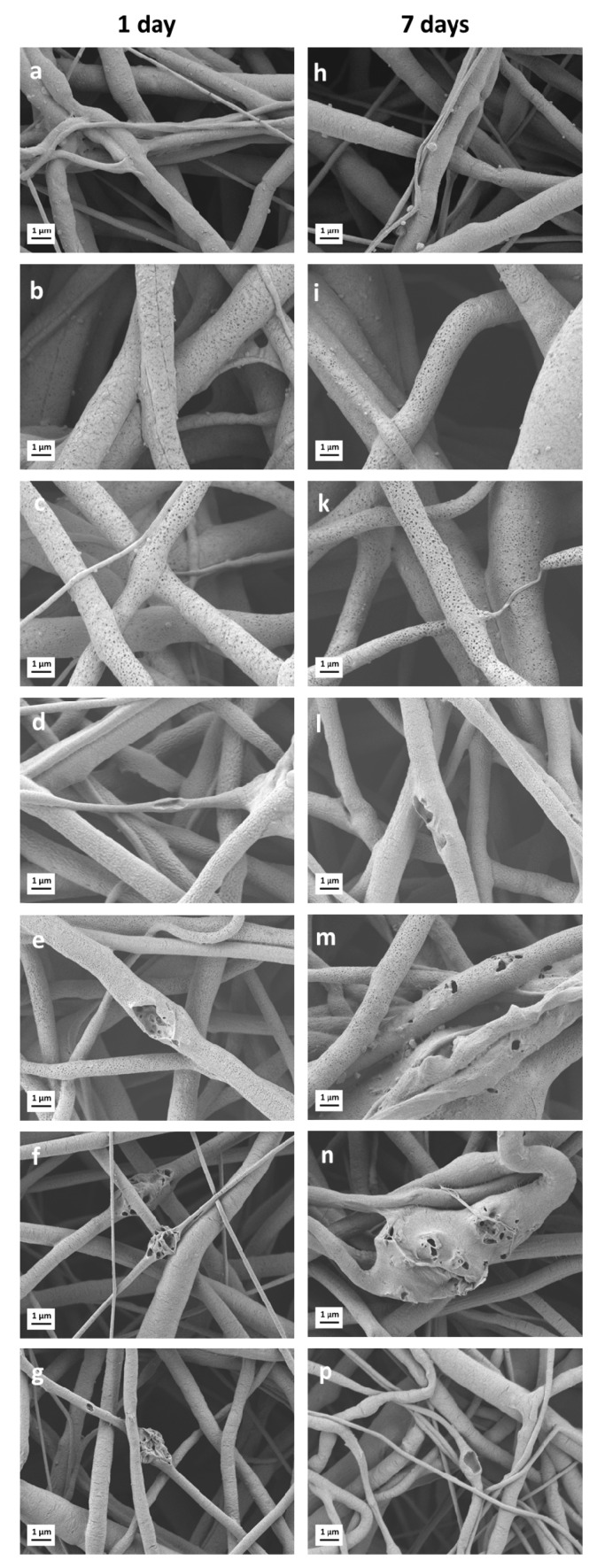
SEM images of electrospun fiber mats after immersion in PBS solution: (**a**,**h**) neat PCL (S5); (**b**,**i**) PCL/PGS_p_ (S1); (**c**,k) PCL/PGS_mxl_ (S2); (**d**,**l**) PCL/PGS_mxl_/13-93 (S3); (**e**,**m**) PCL/PGS_mxl_/13-93BS (S4); (**f**,**n**) PCL/13-93 (S6) and (**g**,**p**) PCL/13-93BS (S7), respectively.

SEM micrographs of PCL/PGS_p_ and PCL/PGS_mxl_ after 1 and 7 days of immersion in PBS are reported in Figure 5b,i and Figure 5c,k, respectively. They clearly indicate that fiber degradation takes place already after 1 day of immersion. According to the literature, PGS degradation takes place via surface erosion [26,56]. The occurrence of the latter phenomenon is manifested by the formation of the porous structure and polymer leaching observed in samples, for example in Figure 5b,c.

Samples containing neat PCL (Figure 5a,h) did not exhibit, even after 7 days of immersion, significant evidence of degradation confirming the results of the pH measurements. On the other hand, Figure 4 shows that blends containing both PGS polymer and BG particles, PCL/PGS_mxl_/13-93 and PCL/PGS_mxl_/13-93BS, which in contact with PBS did not promote acidification of the medium. In contrast, the pH increased after 4 h of immersion, reached a maximum value after 96 h and slightly dropped after 168 h. This effect could be ascribed to the alkaline dissolution from the BG, which compensates the acidification effect caused by PGS. This effect of ion release from BGs, counteracting acidic degradation effect of polyesters, has been reported [57].

Additionally, in the samples containing only PCL and BGs, PCL/13-93 and PCL/13-93BS, pH increased due to BG dissolution and alkaline ion release. Correspondingly, SEM micrographs of PCL/PGS_mxl_/13-93, PCL/PGS_mxl_/13-93 BS, PCL/13-93, and PCL/13-93 BS evidenced huge pores, indicating the dissolution of BG particles (Figure 5f,n,g,p). 

### 3.5. In Vitro Acellular Bioactivity

**Figure 6:**
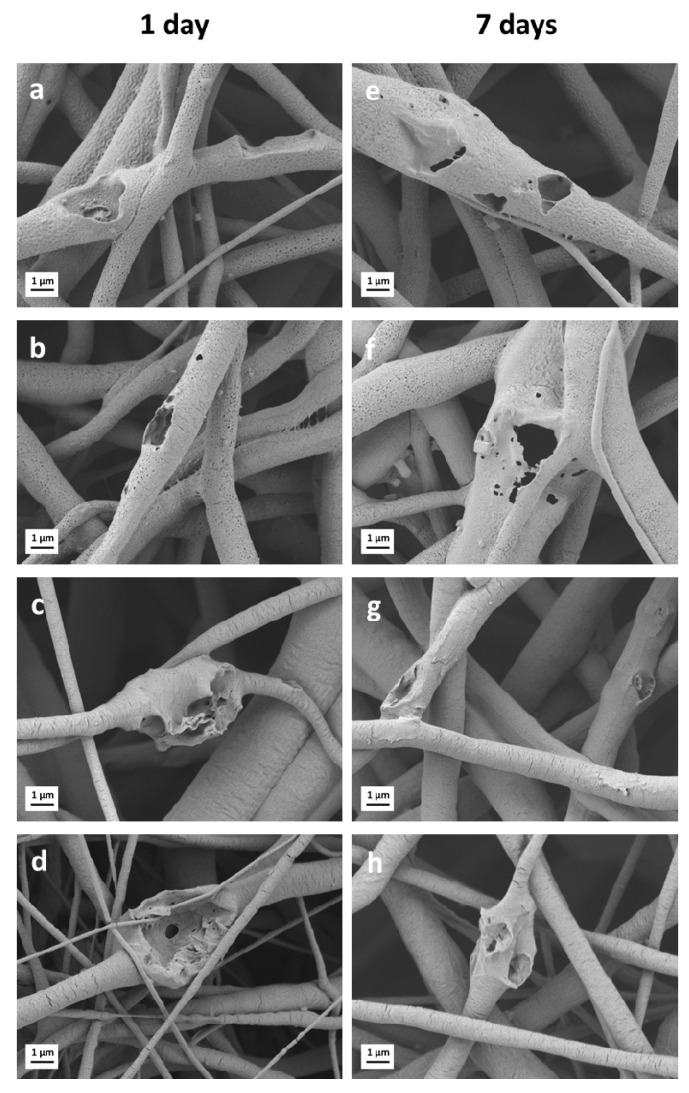
SEM images of electrospun fiber mats containing bioactive glass particles after immersion in SBF solution: (**a**,**e**) PCL/PGS_mxl_/13-93 (S3); (**b**,**f**) PCL/PGS_mxl_/13-93BS (S4); (**c**,**g**) PCL/13-93 (S6); (**d**,**h**) PCL/13-93BS (S7).

To assess the ability of the fabricated samples to form hydroxyapatite on their surface, which is a marker of bioactivity, electrospun composite mats were immersed in the SBF solution [44]. Blended fibrous mats were used as a control. Representative SEM micrographs of the composite mats containing bioactive glass particles after soaking in SBF for 1 and 7 days are reported in Figure 6. No biomineralization effect was observed. Indeed, after one day of immersion, BG particles were already released from the fibers, as evidenced by the pores left on their surface, similarly to the results shown previously using PBS. Considering the target on soft tissue engineering applications, as for example wound healing, previous study [58] reported that the higher potential and role of the BG in wound healing efficacy is represented by controlled delivery of ions, regardless their mineralization capability. 

Degradation of PGS via surface erosion can also be detected in Figure 6e,f when considering PCL/PGS_mxl_/13-93 and PCL/PGS_mxl_/13-93BS systems, respectively. 

### 3.6. Mechanical Properties

**Figure 7:**
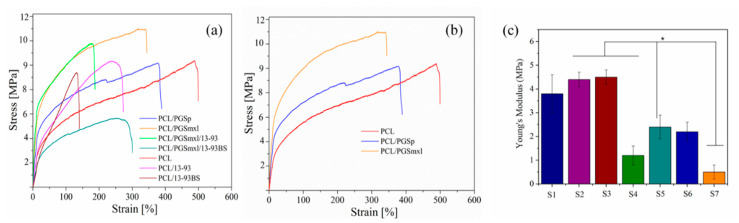
Representative stress–strain curves of as spun samples: (**a**) blended and composite fibers; (**b**) only blended fibers (without BG particles). Young’s Modulus values for all samples (Sample labels are reported in Table 1) (* *p* < 0.05). In Figure 7 (**c**) sample labels are reported accordingly to Table 1.

It is known that to ensure structural integrity and provide an adequate support to cells, mechanical properties of biodegradable fiber scaffolds should match those of the regenerating tissues. In the present study, uniaxial tensile tests were carried out to evaluate the mechanical properties of the as-spun fiber mats under external stress. Resulting stress–strain curves as well as averaged values of Young’s modulus, ultimate tensile strength and failure strain are reported in Figure 7a,b and Table 3, respectively. The stress–strain profiles of all tested fiber mats are consistent with the profiles typical of elastomeric materials [8]. 

The obtained values of the Young’s modulus (E) for PCL specimens (2.4 ± 0.5 MPa) were found to be lower than previously published data [10,47]. On the other hand, values of E for PCL/PGS blends (3.8 ± 0.8 MPa and 4.4 ± 0.5 MPa for PCL/PGS_p_ and PCL/PGS_mxl_, respectively) are close to values reported in the literature [28,47]. E of PCL/PGS_p_ and PCL/PGS_mxl_ blends significantly increased in comparison to that of neat PCL samples, as reported in Figure 7c, indicating an improvement of scaffolds stiffness (Figure 7b) with the introduction of PGS. Furthermore, as reported in Table 3, UTS of PGS/PCL samples was found to be comparable, while values of failure strain decreased when PCL/PGS blends were considered. Those results could be related to the higher average fiber diameter of all blended PCL/PGS samples, respect to the neat PCL. In fact, the mechanical properties of fiber scaffolds were observed to depend on fiber diameter and average fiber diameter distribution. As seen in Figure 7a, the mechanical properties of the samples containing BG particles were found to be lower than those of BG-free fibers. This feature can be ascribed to the increased inhomogeneity in the distribution of the average fiber diameter as well as to the presence of a weak interface between BG particles and polymer, as previously reported by Liverani et al. [43]. The weak interface can be observed in the SEM micrographs of the samples after the degradation and bioactivity test (Figure 5 and Figure 6), in fact from the signs left from the BG particles, it is possible to assume superficial and weak incorporation of the particles in the polymeric fibers. This reduction of mechanical properties is interestingly more visible in the fibers containing borosilicate glass particles. Therefore, the addition of BGs particles of different composition is meant to add functionalities related to ion release, but such different types of particles has also an effect on the resultant mechanical properties of the fibers.

### 3.7. Cell Study

**Figure 8:**
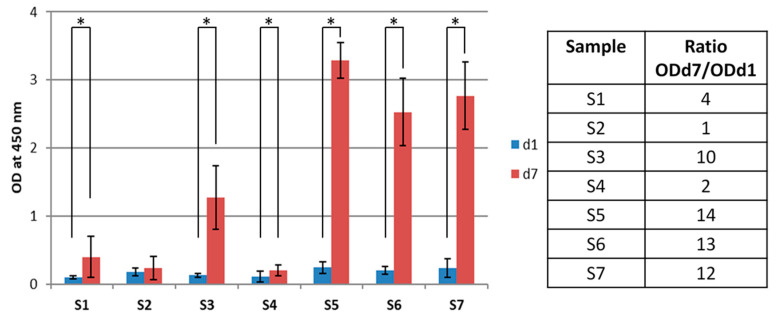
WST-8 analysis: optical density at 450 nm for all samples 1 and 7 days after the seeding. (Sample labels are reported in Table 1) (* *p* < 0.05). A table reporting the increase of the OD values (at 450 nm) expressed as ratio between the measured OD 7 days after seeding respect to the OD value at 1 day. Sample labels are reported accordingly to Table 1.

The stromal cell line ST-2 was used to evaluate cell viability, proliferation, and adhesion to fiber mats. The results of WST-8 assay after 1 and 7 days of incubation are presented in Figure 8. It can be noticed that cell viability is significantly lower in all samples containing PGS, i.e., PCL/PGS_p_, PCL/PGS_mxl_ and PCL/PGS_mxl_/13-93, and PCL/PGS_mxl_/13-93BS, as compared to neat PCL control. This outcome is in contrast to previous research, where an improved cell adhesion and proliferation by PCL/PGS fiber mats compared to PCL fiber mats was reported [13,20,47]. Such discrepancy could be explained as follows. According to the literature above, samples immersion in aqueous 70% *v/v* ethanol solution is usually utilized for disinfection purposes prior the cell seeding stage. Most likely, this procedure is aimed not only to disinfect samples but also to remove unreacted monomers and oligomers from the PGS structure. Indeed, the presence of residual monomers can strongly affect the pH of culture medium and be harmful for cells. In this regard, previous works [8,59] studied the possible effects of the disinfection procedures (using of 70% *v/v* aqueous ethanol versus UV light treatment) on the chemical composition and morphology of as-spun PCL/PGS mats. Results arising from these studies indicate that sample immersion in ethanol leads, even after only 1-h soaking, to the complete removal of PGS from the blend. Authors reported that leaching of PGS is accompanied by the formation of pores on the fiber surfaces. In the present study, UV light for 1 h was used to disinfect the samples. Then, pre-conditioning in culture medium was performed for 1 min just before the cell seeding. 

Results of WST-8 assay for the blended and composite fiber mats presented in Figure 8 show no significant difference after 1 day from the seeding in cell viability with respect to neat PCL. 

**Figure 9:**
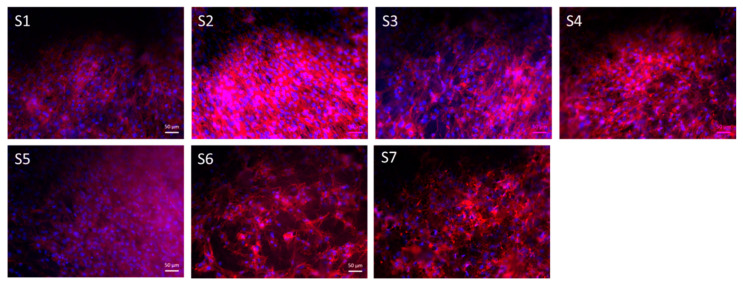
Fluorescence images of ST-2 cells on as-spun fiber mats after 7 days of incubation. (Sample labels are reported in Table 1).

However, after 7 days, it is possible to detect a significant increase in the measured OD values for all samples, beside PCL/PGS_mxl_, whose normalized ratio values (d7)/d1 are reported in Figure 8. This ratio was obtained dividing the average OD value for d7 (ODd7) by the average OD value for d1 (ODd1) for each sample. The observed increase is different depending on the specific system considered. As already noticed from the pH measurements during the degradation test, it is clear that the presence of PGS and its related fast degradation rate affect not only cell adhesion but also their proliferation. This effect appears to be mitigated by the presence of 13-93 BG particles, while the same result is not detectable in the sample containing 13-93BS, confirming that the BG degradation rate and the ion release profile can be different and tailored depending on BG composition. On the other hand, from the results obtained on the electrospun fibers without PGS and reported in Figure 8 and Figure 9, it is possible to notice that the addition of BG particles did not affect neither the cell adhesion nor proliferation. Furthermore, the two BG compositions showed comparable performance.

As already reported in literature [8], the PGS degradation from the surface of the electrospun fibers could reduce the cell adhesion capability, because of a local effect determined by those acidic degradation products. Therefore, a reduced number of cells could be detected on blended PCL/PGS, as reported in Figure 8. From the fluorescence microscopy study performed 7 days after seeding, it can be noticed that the stained living cells look elongated (as indicated by actin filaments in red) on the scaffolds in all sample types, as shown in Figure 9. The differences between samples S1 and S2 could be related to the fact that the PGS prepolymer (not crosslinked) was already completely degraded at the time point and the recorded effect on cell proliferation is mainly due to PCL, even though the number of adherent cells was less than that of the control (as reported in Figure 8 from WST-8 test at d1). Moreover, the PGS_mxl_ component was not fully degraded and the presence of degradation products could be responsible for the reduced number of cells detected, as reported in Figure 9.

## 4. Conclusions

Homogeneous bead-free PCL/PGS fiber mats were prepared in this work by electrospinning. Bioactive glass particles of two different BG compositions, 13-93 and 13-93BS, were also successfully incorporated within the electrospun mats. The effect of fiber composition on the mechanical properties and degradation characteristics was also assessed. In particular, PGS prepolymer improved scaffold’s stiffness, while the incorporation of BG particles led to the overall decrease of mechanical properties. From pH measurements, it could be assumed that ionic dissolution species from BGs compensated the presence of acidic degradation products from PGS prepolymer. Moreover, it was noticed that BG particles could be completely released from the composite fibers already after 1 day of immersion in SBF, offering a controlled particle release, suitable for several applications where the release of therapeutic ions from BGs plays a pivotal role. Additionally, the absence of mineralization revealed the suitability of the obtained fiber mats for such applications where mineralization is not required, for example in wound healing. The promising results form the basis for further comparative studies in which different BG compositions could be considered in particulate form for incorporation in PCL/PGS electrospun fibers.

## Figures and Tables

**Table 1:** Composition of poly(epsilon caprolactone) (PCL) and poly(epsilon caprolactone)/poly(glycerol-sebacate)PCL/PGS polymeric solutions and parameters for electrospinning of PCL and PCL/PGS fiber mats.

Sample Name	Label	Solution Concentration [% *w/v*]/Polymer-BG Ratio PCL:PGS:BG	Applied Voltage [kV]	Distance Tip-Target [cm]	Needle Diameter	Flow Rate [mL/h]	Temperature [^o^C]	Relative Humidity [%]
PCL/PGS_p_	S1	20/1:0.5:0	15	11	21G	0.4	24 ± 0.8	34 ± 12
PCL/PGS_mxl_	S2	20/1:0.5:0	15	11	21G	0.4	24 ± 0.8	34 ± 12
PCL/PGS_mxl_/ 13-93	S3	20/1:0.5:0.3	15	11	18G	0.4	24 ± 0.8	34 ± 12
PCL/PGS_mxl_/ 13-93BS	S4	20/1:0.5:0.3	15	11	18G	0.4	24 ± 0.8	34 ± 12
PCL	S5	20/1:0:0	15	11	21G	0.4	24 ± 0.8	34 ± 12
PCL/13-93	S6	20/1:0:0.3	15	11	18G	0.4	24 ± 0.8	34 ± 12
PCL/13-93BS	S7	20/1:0.5:0.3	15	11	18G	0.4	24 ± 0.8	34 ± 12

**Table 2:** Composition in wt.% of the synthesized bioactive glasses (BGs) used for the composite fibers.

Bioactive Glass Denomination	SiO_2_	B_2_O_3_	CaO	K_2_O	Na_2_O	MgO	P_2_O_5_
13-93	56.6	-	18.5	11.1	5.5	4.6	3.7
13-93BS	20	36.6	18.5	11.1	5.5	4.6	3.7

**Table 3:** Average fiber diameter and mechanical properties of electrospun fiber mats.

Sample Name	Sample Label	Average Fiber Diameter [μm]	Young’s Modulus [MPa]	Ultimate Tensile Strength (UTS) [MPa]	Failure Strain [%]
PCL	S5	0.9 ± 0.4	2.4 ± 0.5	1.3 ± 0.2	447 ± 226
PCL/PGS_p_	S1	1.5 ± 0.5	3.8 ± 0.8	1.0 ± 0.2	219 ± 112
PCL/PGS_mxl_	S2	1.5 ± 0.6	4.4 ± 0.3	1.2 ± 0.2	200 ± 103
PCL/PGS_mxl_/13-93	S3	1.6 ± 0.7	4.5 ± 0.3	1.0 ± 0.1	117 ± 62
PCL/PGS_mxl_/13-93BS	S4	1.7 ± 0.9	1.2 ± 0.4	0.6 ± 0.1	185 ± 106
PCL/13-93	S6	1.1 ± 0.7	2.2 ± 0.4	1.1 ± 0.2	228 ± 139
PCL/13-93BS	S7	1.1 ± 0.7	0.5 ± 0.3	0.9 ± 0.2	115 ± 57
